# Platelets-related signature based diagnostic model in rheumatoid arthritis using WGCNA and machine learning

**DOI:** 10.3389/fimmu.2023.1204652

**Published:** 2023-06-23

**Authors:** Yuchen Liu, Haixu Jiang, Tianlun Kang, Xiaojun Shi, Xiaoping Liu, Chen Li, Xiujuan Hou, Meiling Li

**Affiliations:** ^1^School of Clinical Medicine, Peking Union Medical College, Beijing, China; ^2^Peking Union Medical College Hospital, Chinese Academy of Medical Sciences and Peking Union Medical College, Beijing, China; ^3^Department of Rheumatology, Dongfang Hospital, Beijing University of Chinese Medicine, Beijing, China; ^4^Department of Rheumatology, Fangshan Hospital Beijing University of Chinese Medicine, Beijing, China; ^5^Department of Rheumatology, Fuyang Hospital of Anhui Medical University, Fuyang, Anhui, China; ^6^Department of Rheumatology, The Second Affiliated Hospital of Guangxi Medical University, Nanning, Guangxi, China

**Keywords:** rheumatoid arthritis, machine learning (ML), diagnostic model, platelet, bioinformatics analysis

## Abstract

**Background and aim:**

Rheumatoid arthritis (RA) is an autoinflammatory disease that may lead to severe disability. The diagnosis of RA is limited due to the need for biomarkers with both reliability and efficiency. Platelets are deeply involved in the pathogenesis of RA. Our study aims to identify the underlying mechanism and screening for related biomarkers.

**Methods:**

We obtained two microarray datasets (GSE93272 and GSE17755) from the GEO database. We performed Weighted correlation network analysis (WGCNA) to analyze the expression modules in differentially expressed genes identified from GSE93272. We used KEGG, GO and GSEA enrichment analysis to elucidate the platelets-relating signatures (PRS). We then used the LASSO algorithm to develop a diagnostic model. We then used GSE17755 as a validation cohort to assess the diagnostic performance by operating Receiver Operating Curve (ROC).

**Results:**

The application of WGCNA resulted in the identification of 11 distinct co-expression modules. Notably, Module 2 exhibited a prominent association with platelets among the differentially expressed genes (DEGs) analyzed. Furthermore, a predictive model consisting of six genes (MAPK3, ACTB, ACTG1, VAV2, PTPN6, and ACTN1) was constructed using LASSO coefficients. The resultant PRS model demonstrated excellent diagnostic accuracy in both cohorts, as evidenced by area under the curve (AUC) values of 0.801 and 0.979.

**Conclusion:**

We elucidated the PRSs occurred in the pathogenesis of RA and developed a diagnostic model with excellent diagnostic potential.

## Introduction

Rheumatoid arthritis (RA) is a significant public health concern worldwide, affecting a considerable proportion of the population. It is a chronic autoimmune disease characterized by inflammation of the joints, which causes pain, stiffness, and can lead to severe disability in some individuals (1). The prevalence of RA is estimated to be around 0.56% globally (2). Despite extensive research efforts, the pathogenesis of RA remains incompletely understood, with complex interactions between genetic, environmental, and immunological factors likely playing a role in its development (3, 4). Furthermore, current diagnostic methods for RA are still suboptimal, highlighting the need for reliable and efficient biomarkers to improve patient outcomes (5).

One area of interest in RA research has been the role of platelets in the disease’s pathogenesis (6–8). Platelets play a crucial role in the autoimmune process and have been shown to be closely associated with inflammatory markers and disease activity in RA (9, 10). Various molecules derived from platelets, such as interleukin-1 (IL-1), transforming growth factor-β (TGFβ), and CXCL4/7, have been identified as potent mediators of RA pathogenesis (11–13). However, while platelet levels have been investigated as a potential biomarker for RA diagnosis, current evidence suggests that this approach has limited diagnostic utility (14). Therefore, there is an unmet need to explore new biomarkers for RA diagnosis and prognosis.

As a typical autoinflammatory disease, the study of less well-studied systems and cell types could help build a more comprehensive clinical picture of RA progression and identify new biomarkers. Thus, our study aimed to elucidate platelet-related signatures (PRS) regulation using an integrated bioinformatic analysis of a publicly available mRNA database. The application of machine learning methods has been a focus of various medical areas (15). The development of technology provides the possibility of developing a new effective diagnostic model for RA (16). Although limited studies have focused on applying machine-learning methods to RA patients recently (17, 18), our study is the first use of a machine-learning approach to develop a diagnostic model based on the platelet-related pathway. LASSO (Least Absolute Shrinkage and Selection Operator), an unsupervised machine learning method, offers superior performance in building diagnostic models by performing simultaneous feature selection and regularization, efficiently identifying the most relevant predictors. This quality reduces model complexity and overfitting, leading to more accurate and generalizable diagnostic predictions. This approach holds the promise of identifying novel biomarkers and improving RA diagnosis and management.

While RA’s exact pathogenesis remains unclear, platelets have been extensively studied as potential contributors to the disease’s development. Our study aims to contribute to this growing body of research by exploring the regulation of PRS using an integrated bioinformatic approach and developing a diagnostic model based on platelet-related pathways. Our findings could provide new insights into RA pathogenesis and lead to the discovery of novel biomarkers for improved diagnosis and treatment outcomes.

## Methods

### Study design and datasets collection

In this study, bioinformatic analysis was used to identify the PRS in RA. We first achieved the DEGs in the exploration cohort. Then we used WGCNA to identify the co-expressed modules and further used functional enrichment and GSEA to elucidate the platelet-related module. We then used the LASSO algorithm to develop a diagnostic model, we then validated its diagnostic efficiency in both the exploration cohort and an outside validation cohort. In order to further explore the biological relationship of the PRS model, we used ssGSEA to explore the correlation of our PRS model and the immune infiltration.

We obtained RA patients’ transcriptome and clinical information from the GEO database (https://www.ncbi.nlm.nih.gov/geo/). The two cohorts (GSE93272 (19) and GSE17755 (20)) were included in our study. Both datasets contain peripheral blood cell samples. The basic information of the two datasets is concluded in **Table 1**.

**Table 1:** General information of obtained datasets.

Datasets	Platform	Sample	RA samples	Control samples
GSE93272	GPL570	275	232	43
GSE17755	GP1291	165	112	53

### Differential expressed gene and WGCNA analysis

We used the ‘limma’ package from R to assess RA patients’ differential expressed genes (DEGs) and healthy controls in GSE93272. DEGs were defined as |log2FC|>1 (FC, fold change) and adj.P<0.05.

We then performed WGCNA analysis by the ‘WGCNA’ package in R to assess the biological co-expressing network in the DEGs (21). Our WGCNA parameters were networkType = “unsigned”, minModuleSize = 20, mergeCutHeight = 0.25 and deepSplit = 2.

### Enrichment analysis and module identification

GO and KEGG analysis was performed by ‘cluster profiler’ in R to identify the platelets-related module (22). We screened for the module with the closest relationship to the platelet.

### Gene set enrichment analysis (GSEA)

The GSEA analysis was performed to show whether the defined gene set’s performance showed a significant difference in RA patients and healthy controls (23). To determine the role of platelets in the pathogenesis of RA, the enrichment of GOBP PLATELET ACTIVATION and GOBP PLATELET AGGREGATION between RA patients and healthy controls was illustrated *via* the ‘clusterProfiler’ package of R (22).

### Development of diagnostic model *via* machine learning

We used the LASSO algorithm to construct a diagnostic model; the ‘glmnet’ package was used to train our model. After removing genes with coefficients of 0, we used the remaining coefficients to construct the diagnostic model. The formula risk is shown as follows: risk score =(ExpressionGENE1 × CoefficientGENE1) + (ExpressionGENE2 × CoefficientGENE2) + ⋯+(ExpressionGENEn × CoefficientGENEn). The box plot is applied to display the risk score of the individuals.

### Validation of diagnostic potential of PRS signature in both cohorts

We tested the diagnostic ability of our diagnostic model in the testing cohorts *via* the ‘ROCR’ package of R. We evaluated its diagnostic potential in GSE93272 and an outside validation group, GSE17755. The area under the curve (AUC) is calculated to display its diagnostic potential.

### Single-sample gene set enrichment analysis

To further analyze the biological function of PRS signature, we performed ssGSEA to analyze the infiltration score of 16 immune cells and the activity of 13 immune pathways. The ‘gsva’ package was used to perform the ssGSEA in GSE93272 (24). The c5.all.v7.0.symbols.gmt gene set was selected as the reference gene set.

### Statistical analysis

All statistical analyses were performed *via* R (ver. 4.0.2). P<0.05 was considered statistically significant.

## Results

### Identification of DEGs and platelets-related genes

**Figure 1:**
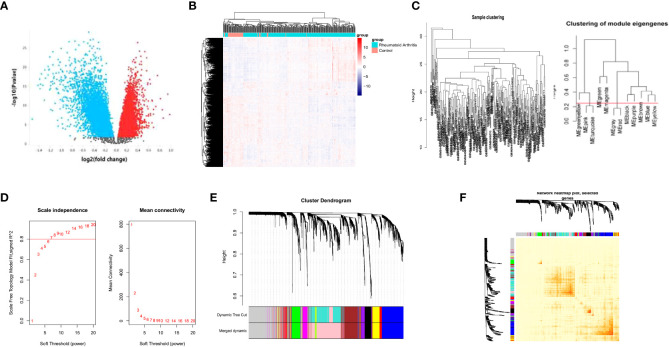
WGCNA analysis of the DEGs. Volcano plot of the DEGs between RA patients and healthy controls **(A)**. heatmap of gene expression in GSE93272 **(B)**. Sample and module dendrogram of GSE93272 **(C)**. Analysis of scale-free index and mean connectivity of various soft thresholds, the red line indicating the selected soft threshold **(D)**. Cluster dendrogram **(E)** and topological overlap matrix **(F)** of all DEGs.

We achieved the gene expression matrix and the clinical data of GSE93272 after data preprocessing through the ‘GEOquery’ and ‘limma’ packages of R. The DEG selecting criteria were log2|FC|≥1 and adj.P ≤ 0.05. (FC, fold change; adj.P: adjusted P value). We obtained 3776 upregulated DEGs and 4714 downregulated DEGs (**Figure 1A**). The expression of the DEGs in each sample is presented in **Figure 1B**.

### Identification of PRS through WGCNA

We used a sample clustering tree to elucidate the outliers (**Figure 1C**). We then selected the soft threshold β by the “pickSoftThreshold” function in the WGCNA (**Figure 1D**) and identified the modules (**Figure 1C**). The soft threshold was set as seven. We further developed a hierarchical clustering tree, each branch representing genes with similar expression and biological functions (**Figure 1E**). Moreover, we analyzed the interaction between elucidated modules by calculating the degree of connectivity (**Figure 1F**).

### Enrichment analysis of the module

**Figure 2:**
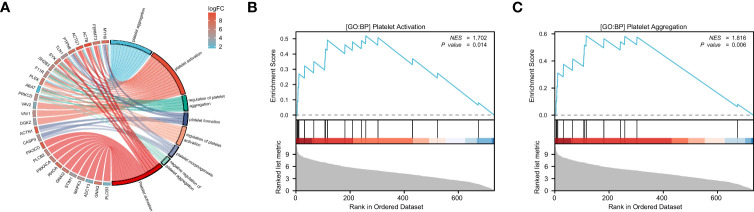
The enrichment analysis and GSEA of module 2. GO and KEGG analysis of module 2, the color represents the log|FC| of the pathway **(A)**. GOBP PLATELET ACTIVATION **(B)** and GOBP PLATELET AGGREGATION **(C)** are significantly enriched in RA patients compared to the healthy controls.

We performed GO and KEGG analysis using the ‘clusterProfiler’ package in R to identify the module that has the closest relationship to platelets, which is referred to as module 2. Subsequently, we conducted GO and KEGG analysis on module 2 to identify the platelet-related pathway (**Figure 2A**). The genes involved in this pathway shown in **Figure 2A** were identified as potential PRS candidates. To ascertain the activity of platelet-related pathways more accurately, we employed GSEA. Specifically, we evaluated the activity of GOBP PLATELET ACTIVATION (**Figure 2B**) and GOBP PLATELET AGGREGATION (**Figure 2C**) for module 2.

### Construction of the PRS model

**Figure 3:**
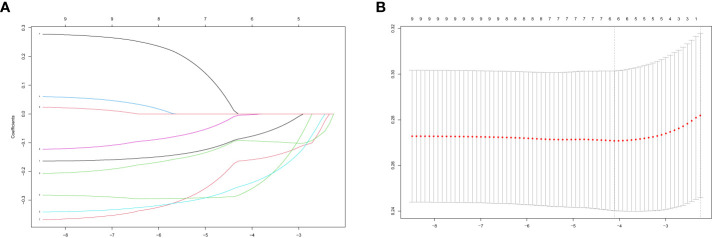
Performing LASSO algorithm. Coefficient profile plots of each independent variable **(A)**. Partial likelihood deviance for LASSO logistic regression **(B)**.

We acquired the expression data of candidate PRS from GSE93272 as a training group. We then utilized the LASSO algorithm to derive the coefficient profile plots (**Figure 3A**) and partial likelihood deviance (**Figure 3B**). From these analyses, we identified six non-zero coefficient signatures, namely MAPK3, ACTB, ACTG1, VAV2, PTPN6, and ACTN1, which were used to construct the risk score model. The formula for the risk score is as follows: riskScore =0.08×Expression_MAPK3 _+ 0.16×Expression_ACTB_ +0.27×Expression_ACTG1 _+ 0.25×Expression_VAV2 _+ 0.003×Expression_PTPN6 _+ 0.09×Expression_ACTN1_.

### Evaluation of diagnostic potential in training and validation cohorts

**Figure 4:**
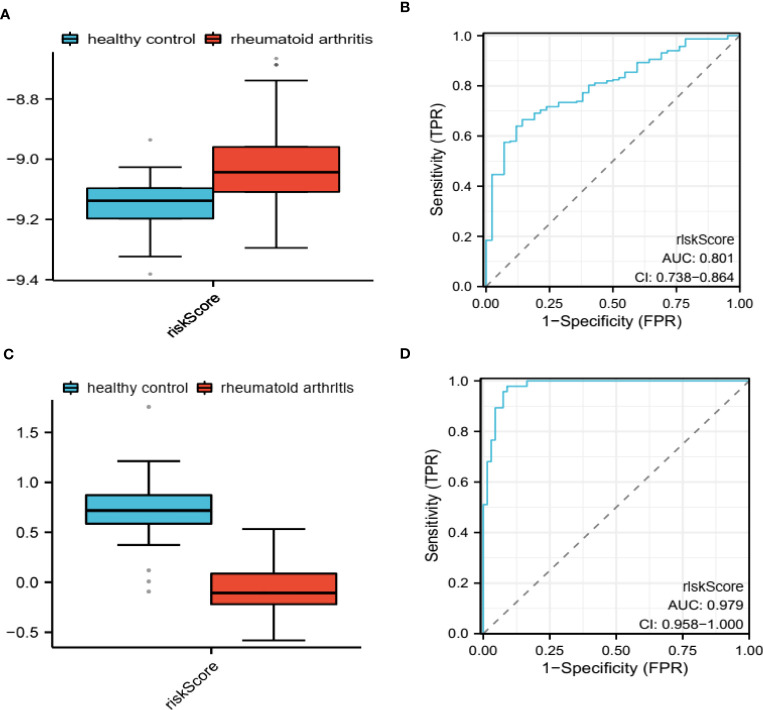
Evaluation of diagnostic potential in GSE93272 and GSE17755. PRS-based riskScore calculated in GSE93272 **(A)** and GSE17755 **(C)**. ROC for evaluating the diagnostic potential of PRS in GSE93272 **(B)** and GSE17755 **(D)**.

We assessed the predictive capability of our PRS in both the training and validation groups by computing the risk score for each sample in these cohorts (**Figures 4A, C**). We subsequently employed the ROC analysis to determine the diagnostic potential of our model. The AUC values were 0.801 (**Figure 4B**) and 0.979 (**Figure 4D**) for the training and validation groups, respectively, indicating excellent diagnostic accuracy for both cohorts. We have also applied the Precision-Recall curve through ‘modEVA’of R to further evaluate its efficiency (**Supplementary Figure 1**). To distinguish between High- and Low-risk groups, we used a cutoff value of 9.08 in the training group.

### Analysis of immune-related status and genes

**Figure 5:**
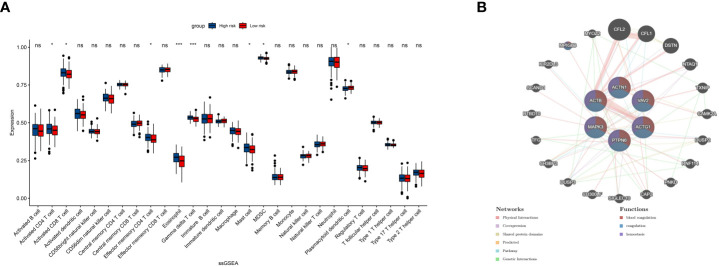
ssGSEA shows the infiltration result of 13 immune cells and 16 immune pathways **(A)**. Twenty top related genes to the 6 PRS and their common pathways **(B)**. ns, insignificant.*P<0.05,***P<0.00005.

To further examine the immune status associated with the PRS, we utilized single-sample Gene Set Enrichment Analysis (ssGSEA) to analyze the immune landscape (**Figure 5A**). Our findings indicate that activated CD4/8 T cells, effector memory CD4 T cells, Eosinophils, Gamma delta T cells, Mast cells, Myeloid-derived suppressor cells (MDSCs), and plasmacytoid dendritic cells are significantly different between the High- and Low-risk groups. Additionally, we employed GeneMANIA to identify the top 20 genes most closely related to the PRS (**Figure 5B**). Furthermore, we examined the co-expression network of the PRS, which revealed a strong association with blood coagulation, coagulation, and hemostasis.

## Discussion

RA is an autoimmune disorder involving multiple systems. The current first-line diagnosis criteria for RA is the 2010 ACR/EULAR classification criteria (25). The diagnosis based on this requires a combination of clinical performance and laboratory results. However, this meant the trauma caused by RA already existed by the time of diagnosis. Thus, developing a new bio-signature could enhance the diagnosis of RA and contribute to its clinical outcome. Platelets are deeply involved in regulating the innate immune system (26). Elevated platelet indices have been associated with RA disease activity (27, 28). Platelets are believed to be involved in the inflammatory process through a direct link with immune cells or the secretion of inflammatory mediators (29, 30). Platelets have shown a potential for treatment targeting (31). Given the role of platelets in RA, developing a new bio-signature that includes platelet indices could help improve the diagnosis of RA. This approach could allow for earlier detection of the disease, which would enable medical practitioners to initiate treatment strategies early enough to prevent severe damage from occurring. Moreover, targeting platelets could provide a new therapeutic avenue for treating RA.

Our study obtained 3776 upregulated DEGs and 4714 downregulated DEGs by comparing genes expressed in RA and healthy control samples. We then used WGCNA analysis to identify the co-express modules. GO enrichment analysis of each module identifies the module and the genes with the closest interaction with platelets. We then used GSEA to elucidate the activity of pathway GOBP PLATELET ACTIVATION and GOBP PLATELET AGGREGATION and identify 27 candidate PRS. We then used the LASSO algorithm to construct a 6 PRS model that showed excellent diagnostic potential between RA and healthy control in GSE93272 and GSE17755. We divided our samples into the high- and low-risk group based on our PRS and performed ssGSEA to illustrate the immune infiltration landscape. In addition, we used GeneMANIA to demonstrate the related genes and pathways that share the closest relationship with PRS.

In our study, we aimed to identify a platelet-related gene signature that could serve as a potential diagnostic tool for RA. To achieve this goal, we first compared the genes expressed in RA and healthy control samples using RNA sequencing analysis. As a result, we identified 3776 upregulated differentially expressed genes (DEGs) and 4714 downregulated DEGs. We then utilized weighted gene co-expression network analysis (WGCNA) to identify the co-expressed modules and performed gene ontology (GO) enrichment analysis on each module to identify the one with the closest interaction with platelets. Furthermore, we employed gene set enrichment analysis (GSEA) to evaluate the activity of platelet-related pathways and identify 27 candidate PRS genes. Next, we constructed a PRS model using the Least Absolute Shrinkage and Selection Operator (LASSO) algorithm, which showed excellent diagnostic potential between RA and healthy control samples in both GSE93272 and GSE17755 datasets. We used the PRS model to divide the samples into high- and low-risk groups, followed by ssGSEA to illustrate the immune infiltration landscape. Moreover, we analyzed the related genes and pathways closely associated with the PRS using GeneMANIA. Our results demonstrated that the PRS had a close relationship with blood coagulation, coagulation, and hemostasis pathways.

This study found a diagnostic model based on logistic regression of MAPK3, ACTB, ACTG1, VAV2, PTPN6, and ACTN1. MAPK3 is a member of mitogen-activated protein kinase, and participates in signaling cascades that regulate various cellular processes including platelets activation and aggregation (32). The MAPK3 inhibitors have shown the potential to develop a new therapy for RA (33).ACTB, ACTN1 and ACTG1 are members of cytoskeletal proteins. The actin network formation is related to platelet granule release and remodeled upon platelet activation (34). It has also been identified that actin is related to manifestations involving muscle in RA patients (35). VAV2 is a Rho family guanine nucleotide exchange factor (36). It is associated with the platelet-derived growth factor (PDGF) receptor (37). Its protection effect on antigen-induced RA has been verified in an animal model (38). PTPN6 belongs to the protein tyrosine phosphatase family. It is involved in various signaling pathways. PTPNs are in fact found to be critical regulators of both platelet activation and thrombosis (39). Although the biological function of PTPNs in RA has not been clarified, analogs belonging to this family have been identified as risk factors of RA (40, 41).

The study under consideration is the first of its kind to conduct an integrated bioinformatic investigation aimed at exploring signatures associated with platelets in patients suffering from rheumatoid arthritis. This inquiry has allowed us to screen for potential biomarkers through the application of a machine learning method, thus providing crucial insight into the complex and multifaceted nature of this debilitating condition. Subsequently, we have verified the diagnostic efficacy of our proposed Platelet-Related Signature (PRS) in two separate cohorts, confirming its practical utility as a viable tool for clinical assessment and management. These findings represent a significant contribution to the field of rheumatology and may serve as a valuable resource for medical practitioners seeking to improve patient outcomes through more accurate diagnosis and targeted treatment strategies.

While our study presents promising results, several limitations should be acknowledged. Our cohort’s sample size is relatively small, which may limit the generalizability of our findings. Therefore, it is crucial to validate our model in larger cohorts to assess its robustness and reliability. Furthermore, although we have demonstrated the diagnostic potential of our PRS model, it lacks verification from cell- and animal-based experiments. Future research should focus on evaluating the underlying mechanisms of the genes involved in our model through experimentation in laboratory settings. This could include validating the differential expression of candidate genes *in vitro* or using animal models to investigate the functional relevance of these genes in RA pathogenesis. Additionally, other factors such as age, sex, and medication status can influence gene expression patterns in RA patients. In this study, we did not account for the potential confounding effects of these variables. Therefore, future studies should consider including additional clinical and demographic data to better understand the role of these factors in PRS development and interpretation.

In conclusion, while our study highlights the potential of PRS as a diagnostic tool for RA, further research is needed to address the limitations mentioned above and fully evaluate the clinical utility of our approach in the diagnosis and management of RA.

## Data availability statement

Publicly available datasets were also analyzed in this study. This data can be found here: https://www.ncbi.nlm.nih.gov/geo/ under the accession numbers GSE93272 and GSE17755.

## Ethics statement

Ethical review and approval was not required for the study on human participants in accordance with the local legislation and institutional requirements. Written informed consent for participation was not required for this study in accordance with the national legislation and the institutional requirements.

## Author contributions

YL: Conceptualization, Methodology, Software, Formal Analysis, Visualization, Writing - Original Draft. HJ: Data Curation, Writing - Original Draft. TK: Visualization, Software. XS: Software, Validation. XL: Visualization, Writing - Review & Editing. CL: Resources, Supervision. XH: Conceptualization, Funding Acquisition, Resources, Supervision. ML: Conceptualization, Funding Acquisition, Resources, Supervision, Writing - Review & Editing. All authors contributed to the article and approved the submitted version.
