# Supplementary figures and images for: Platelets-related signature based diagnostic model in rheumatoid arthritis using WGCNA and machine learning
Source: Front Immunol. 2023 Jun 23;14:1204652. doi: 10.3389/fimmu.2023.1204652 (PMC10327425; doi:10.3389/fimmu.2023.1204652)

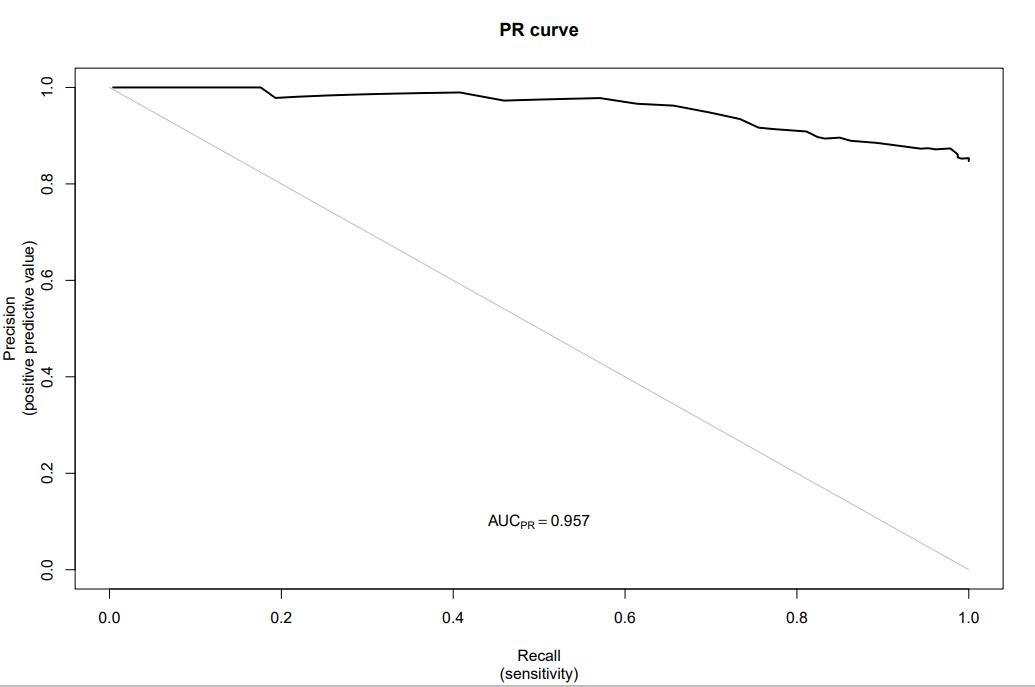


Supplementary figure 1. The precise-recall curve of PRS model in GSE93272

Supplement: Supplementary file 1 [file DataSheet_1.docx]
